# Insulin resistance and dyslipidemia in low-birth-weight goat kids

**DOI:** 10.3389/fvets.2024.1370640

**Published:** 2024-03-26

**Authors:** Huihui Song, Zhuohang Hao, Hehan Feng, Rui Li, Ran Zhang, Sean W. Limesand, Yongju Zhao, Xiaochuan Chen

**Affiliations:** ^1^College of Animal Science and Technology, Southwest University, Chongqing Key Laboratory of Herbivore Science, Chongqing, China; ^2^Yunnan Center for Animal Disease Control and Prevention, Kunming, Yunnan, China; ^3^School of Animal and Comparative Biomedical Sciences, The University of Arizona, Tucson, AZ, United States

**Keywords:** low birth weight, goat, insulin resistance, skeletal muscle, lipid accumulation

## Abstract

Low birth weight (LBW) impairs the development and health of livestock by affecting postnatal growth performance and metabolic health in adulthood. Previous studies on indigenous goats in southwest China showed that LBW goat kids had higher mortality and morbidity rates, including hepatic dyslipidemia and liver damage. However, the mechanism of insulin resistance affecting lipid metabolism under LBW conditions remains unclear. In this study, we conducted *in vivo* glucose-insulin metabolic studies, measured biochemical parameters, and analyzed related regulatory pathways. Both glucose tolerance tests and insulin tolerance tests indicated insulin resistance in LBW goat kids compared to controls (*p* < 0.05). The marker of insulin resistance, homeostasis model assessment (HOMA), was 2.85-fold higher in LBW than in control goats (*p* < 0.01). Additionally, elevated levels of free fatty acids in both plasma and skeletal muscle were observed in LBW goats compared to normal birth weight (NBW) goats (*p* < 0.05). Transcriptome analysis revealed impairments in lipid metabolism and insulin signaling in LBW goats. The observed lipid accumulation was associated with the upregulation of genes linked to fatty acid uptake and transport (*FABP3*), fatty acid oxidation (*PPARA*), triacylglycerol synthesis (*LPIN1* and *DGAT1*), oxidative stress (*ANKRD2*), and insulin resistance (*PGC1*α). Furthermore, the insulin receptor substrate 2 (*IRS2*) was lower in the liver of LBW goat kids (*p* < 0.05). While there was no change in insulin function in skeletal muscle, LBW may lead to lipid accumulation in skeletal muscle by interfering with insulin function in the liver. These findings collectively impact the health and growth performance of livestock.

## 1 Introduction

Adverse environmental conditions and suboptimal fetal growth and nutrition may harm livestock and cause low birth weight (LBW) in animals. According to Barker's Early Origins Hypothesis, adverse conditions *in utero* and early infancy permanently alter the metabolic resetting as an adaptation for survival in a poor environment, increasing the risk of metabolic defects later in life (1). Many studies have shown that LBW animals suffer various metabolic complications, such as insulin resistance, glucose intolerance, dyslipidemia, and pancreatic β-cell dysfunction (2–5). In southwest China, indigenous goat newborns have a higher mortality rate associated with LBW (6), but the impairment of glucose-insulin metabolism in LBW goats remains unclear.

As one of the body's primary tissues, the skeletal muscle plays a pivotal role in numerous biological activities encompassing metabolism and injury defense (7). When lipid accumulation occurs in the skeletal muscle, it prompts the development of conditions such as obesity, sarcopenia, diabetes, and nonalcoholic fatty liver disease (8). In livestock production, intramuscular fat is a key determinant of meat quality, contributing to improved meat shear force and flavor (9). In mouse models, heightened lipid accumulation has been shown to stimulate muscle glucose uptake (10). Maternal obesity in ovine models triggers alterations in the fetal skeletal muscle during the early developmental stages, leading to increased intramuscular adipocytes, fibrosis, and insulin resistance (11). The regulation of lipid accumulation in the skeletal muscle involves various factors, including fatty acid transport and both anabolic and catabolic processes. Notably, FABP4, PLIN1, and DGAT1 are primary regulators in this process. Prior studies have established a robust inverse relationship between the lipid content in the skeletal muscle and insulin sensitivity in animal and human subjects. Increased fatty acids in the skeletal muscle or reduced fatty acid oxidation can lead to insulin resistance (12).

The liver might be one of the first organs to experience insulin resistance because of fetal growth restriction (13). Insulin binds to its receptors and plays a key role in glucose uptake from the blood and storage as glycogen. It also inhibits hepatic gluconeogenesis by preventing the liver from making glucose from other sources, such as amino acids or glycerol. Moreover, insulin regulates hepatic lipid metabolism by stimulating lipid synthesis and storage in the liver and skeletal muscle and inhibiting lipid breakdown and oxidation (14). Our previous study of goat kids showed that LBW leads to hepatic lipid accumulation and dysregulation at 1 month of age (15). However, the evaluation of glucose-regulated insulin metabolism in their postnatal life is still unknown.

In this study, we conducted glucose and insulin tolerance tests to compare glucose homeostasis and examined lipid profiles from plasma samples and the skeletal muscle between normal birth weight (NBW) and LBW goat kids *in vivo*. Furthermore, we investigated the underlying mechanism linking lipid metabolism and identified possible signaling pathways influenced by LBW.

## 2 Materials and methods

### 2.1 Animal ethics and husbandry

All experiments were performed according to the principles and guidelines of the Southwest University Institutional Animal Care and Use Committee (2019, No. GB14925–2010). The Dazu Ruifeng Goat Farm (29° 40′ 55^′′^ N, 105° 31′ 31^′′^ E, Dazu, Chongqing) is surrounded by a blend of shrubs and grasses, maintaining noise levels below 40 dB. Constructed primarily of concrete, the farm is partitioned into separate rooms catering to goats of varying ages. Each room comprised an indoor feeding area and an outdoor playground. The indoor space featured a plastic slatted floor and an iron fence. Furthermore, wooden brooding pens with temperature regulation facilities were provided to accommodate pre-weaned kids indoors (6). Goat kids were selected from the Dazu Ruifeng goat farm and transported to the laboratory at Southwest University at ~4–7 days of age.

Daily mean temperature and mean relative humidity were obtained from the nearby meteorological observatory. There was almost a 2-month period (January and February) when the temperature was < 10°C, and the temperature-humidity index was < 50 in Chongqing (6). In this study, newborns of the pregnant goats from the warm season (temperature between 16–25°C) and cold season (temperature below 11°C) were weighed and designated as normal birth weight (≥ 2.1 kg) or low birth weight ( ≤ 1.89 kg), respectively (6). The definition of LBW was 10% lower than the appropriate weight for gestational age counterparts (16, 17). They were then fed with a milk replacer (BaiNianLongTeng, Yunnan, China) until ~30 days of age. Each 100 g of milk replacer contained 2,113 kJ of energy, 25.5 g of protein, 28.7 g of fat, 36.3 g of carbohydrates, 0.38 g of sodium, and 1.08 mg of calcium. We used 500 ml bottles for manual feeding, 3–4 times a day, along with 100–150 ml of preheated (35–37°C) milk replacer each time.

A total of 18 newborns (control, *n* = 10; LBW, *n* = 8) were selected, and growth performance was measured from 6 to 25 days of age. After *in vivo* studies, goat kids were euthanized at 28–30 days with an overdose of sodium pentobarbital given intravenously. Organs and tissues were dissected, weighed, frozen in liquid nitrogen, and stored at−80°C for further use.

### 2.2 Surgical preparation

**Figure 1:**
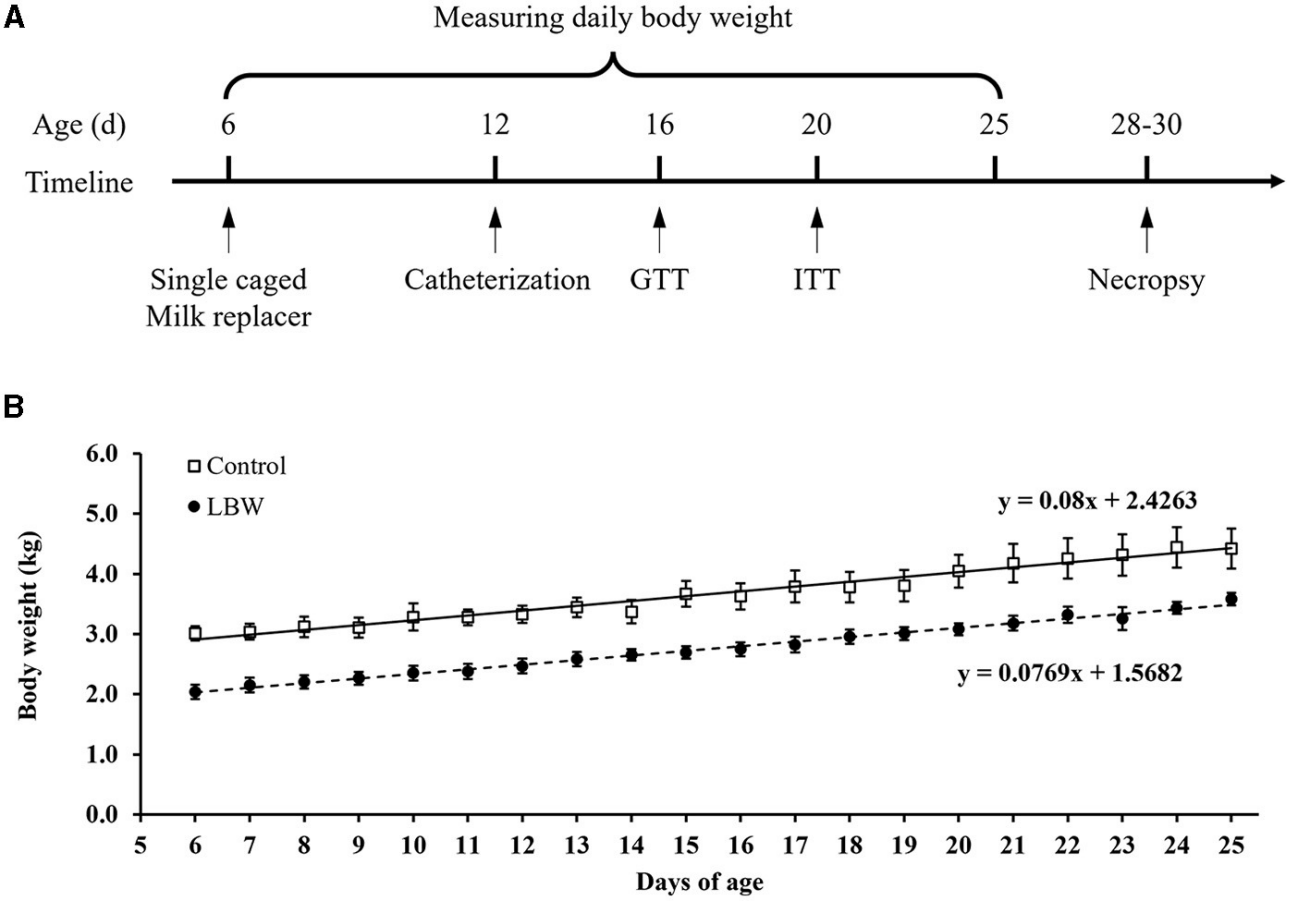
**(A)** Schematic diagram of the experimental design and *in vivo* animal studies. **(B)** Growth rate in newborn goats. GTT, glucose tolerance test; ITT, insulin tolerance test. Birth weight for control (□, *n* = 10) and LBW goat kids (•, *n* = 8) are presented for their 6 to 25 days of postnatal life.

At ~12 days of age, indwelling polyvinyl catheters were surgically placed in the femoral artery and vein for intravenous infusions and blood sampling. Goat kids were anesthetized with an intramuscular injection of xylazine hydrochloride for the surgical procedure (10 ml/kg, Jilin Huamu Animal Health Products, China). After placing catheters in blood vessels, the catheters were tunneled subcutaneously to the flank, exteriorized through a skin incision, and wrapped in a mesh bag secured to the skin. To maintain patency, saline with heparin (50 units/ml) was infused through the catheters daily. The goat kids were allowed to recover for at least 3 days before performing *in vivo* glucose homeostasis studies (Figure 1A).

### 2.3 Intravenous glucose tolerance test

After fasting for 4 h, goat kids were injected intravenously with glucose (50% wt/vol) at 0.5 g/kg of body weight. Arterial blood samples were collected at 0, 15, 20, 25, 40, and 70 min, and the blood glucose concentrations were measured with a glucometer (Performa, Roche, USA). Whole blood samples were collected and centrifuged in EDTA tubes at 4°C at 13,000 rpm for 2 min, and the separated plasma samples were stored at −20°C. Insulin concentrations were measured with radioimmunoassay (Chongqing Medical University, Chongqing, China). The area under the curve of plasma insulin was calculated by Prism 8.0.2 (GraphPad Software, San Diego, CA, USA).

### 2.4 Intravenous insulin tolerance test

After at least a 4-h fast, goat kids were challenged with 0.08 U/kg of recombinant insulin (Humulin R, Lilly, Egypt, intravenous, 0002-8215) (18, 19). Arterial blood samples were collected, and glucose concentrations were measured at 0, 15, 20, 30, 45, 60, 80, and 100 min. The magnitude of the decrease following the insulin tolerance test was determined by the area above the curve. Insulin resistance was estimated by the homeostasis model assessment (HOMA), calculated as HOMA = [fasting insulin (μIU/ml) × fasting glucose (mmol/L)]/22.5 (20).

### 2.5 Biochemical parameters assays

In plasma samples, we assessed the concentration of non-esterified fatty acids employing an enzymatic colorimetric analysis kit (294-63601, Wako, Japan). Furthermore, intramuscular levels of free fatty acids (FFA) (YX-C-B400, SINOBESTBIO, Shanghai, China), triglycerides (YX-C-B408, SINOBESTBIO, Shanghai, China), and glycogen (BC0345, Solarbio, Beijing, China) were quantified by respective commercial kits, and measured by an xMark™ Microplate Absorbance Spectrophotometer (Bio-Rad, Hercules, CA, USA).

### 2.6 RNA sequencing and analysis

It has been found that the number of fibers and the percentage of fiber area of Type I and Type II A in the semitendinosus muscles of young goats are 28–29% (21). RNA quality and integrity were verified by the Northern blot. In the electrophoresis image (Supplementary Figure 1), the left cohort of gel bands was the control, and the right cohort was the LBW group. Semitendinosus tissue samples (control, *n* = 3; LBW, *n* = 4) were randomly selected from each group and sent to Biomarker (Beijing, China) for high throughput RNA sequencing (RNAseq). RNA extraction from the semitendinosus muscle tissue was performed using the miRNeasy Mini Kit (Qiagen, China). To assess the integrity of the RNA, the RNA Nano 6000 Assay Kit and the Agilent Bioanalyzer 2100 system (Agilent Technologies, Santa Clara, USA) were employed.

The sequencing libraries were generated using the NEBNext UltraTM RNA Library Prep Kit (#E7770, New England Biolabs, USA) following the manufacturer's instructions. Index codes were added to associate sequences with each sample, and the cBot Cluster Generation System was employed in accordance with the manufacturer's guidelines, using the TruSeq PE Cluster Kit v4-cBot-HS (Illumina, USA). Following cluster creation, the library preparations were sequenced on an Illumina platform (NovaSeq 6000, USA) to produce paired-end reads. Subsequently, adaptor sequences and low-quality sequence reads were eliminated from the datasets. The raw sequences underwent data processing to yield clean reads. These clean reads were then aligned to the reference genome sequence (ARS1, GenBank assembly accession: GCA_001704415.1) using HISAT2. Only reads with either a perfect match or a single mismatch were subjected to further analysis and annotation based on the reference genome. Gene expression data were normalized using String Tie (22), and the expression levels were quantified as fragments per kilobase of transcript per million mapping reads (FPKM), calculated according to the following formula (23).


$$\begin{array}{l} FPKM=\frac{cDNA\;Fragments}{Mapped\;Fragments\;\left(Millions\right)\;\times \;Transcript\;Length\;\left(kb\right)} \end{array}$$

Differential expression analysis of two samples was performed using edgeR (24). Significantly differential expression was defined by a false discovery rate (FDR) of < 0.05 and |log2(fold change)|≥1.5 (25). Gene functions were annotated based on the Gene Ontology (GO) database (accessed date: 24 February 2020, http://www.geneontology.org/). GO enrichment analysis of the differentially expressed genes (DEGs) was carried out using the GOseq R packages based on Wallenius' non-central hypergeometric distribution (22). GO and Kyoto Encyclopedia of Genes and Genomes (KEGG) terms with corrected at a *p* < 0.05 were considered significantly enriched, distinguishing commonly expressed genes (CEGs) from differentially expressed genes (DEGs) (23).

### 2.7 Quantitative analysis of mRNA expression

We assessed the relative expression levels of differentially expressed genes (DEGs) identified through RNAseq by employing real-time qPCR on semitendinosus muscle samples from two distinct groups, namely the control group (*n* = 6) and the LBW group (*n* = 7). Initially, total RNA was extracted from the semitendinosus muscle tissue using TRIzol Reagent (Thermo Fisher Scientific, Waltham, MA, USA), and the RNA's concentration and purity were determined utilizing a Nanodrop™ One spectrophotometer (Thermo Fisher Scientific, Waltham, MA, USA). To reverse transcribe the mRNA into cDNA, we followed the protocols provided by the manufacturer of the PrimeScript™ RT kit and gDNA Eraser (RR047A, Takara, Beijing, China). The primer sequences are detailed in Table 1. Subsequently, the relative expression of mRNA was quantified using TB Green Premix Ex Taq™ II (RR820A, Takara, Beijing, China) in conjunction with the CFX96 Touch™ Real-time qPCR detection system (Bio-Rad, USA). The real-time qPCR thermal cycling conditions consisted of an initial denaturation step at 95°C for 30 s, followed by 40 cycles at 95°C for 5 s and 60°C for 30 s. For real-time qPCR validation, *GAPDH* was chosen as the internal control. The determination of relative gene expression levels was carried out using the 2^−ΔΔ*Ct*^ method.

**Table 1:** Growth performance and organ weight of goat kids at necropsy.

**Variable**	**Control**	**LBW**	***P*-value**
Body weight, kg	4.82 ± 0.32	3.99 ± 0.19	0.06
Carcass weight, kg	3.54 ± 0.25	2.65 ± 0.10	< 0.05
Brain, g	67.76 ± 2.06	55.49 ± 1.46	< 0.01
Heart, g	33.88 ± 2.62	30.88 ± 1.75	NS
Liver, g	144.82 ± 8.48	136.43 ± 8.90	NS
Lungs, g	87.90 ± 5.99	68.23 ± 2.63	< 0.05

### 2.8 Statistical analysis

A comparison of the growth rate was performed by linear regression using Prism 8.0.2 (GraphPad Software). Data from glucose and insulin time courses were analyzed by repeated measurement with two-way ANOVA. All other data were compared between groups using paired Student's *t*-test. Statistical analysis was performed using SPSS Statistics 19.0 (SPSS Inc., Armonk, NY, USA). Values were expressed as mean ± SEM; a *p* < 0.05 was considered significant.

## 3 Results

### 3.1 Weights and growth characteristics in LBW goat kids

The average body weight of LBW goats at birth (1.87 ± 0.09 kg, *p* < 0.05) was lower than that of goats in the control group (2.52 ± 0.11 kg). At necropsy, LBW body weights were 17% lower than those of the control group (*p* = 0.06), and the carcass, brain, and lungs were significantly lighter in weight in the LBW group (Table 1). The average daily gain from 6 to 25 days of age did not differ between the LBW and control groups (Figure 1B).

### 3.2 Impaired glucose tolerance and insulin insensitivity

**Figure 2:**
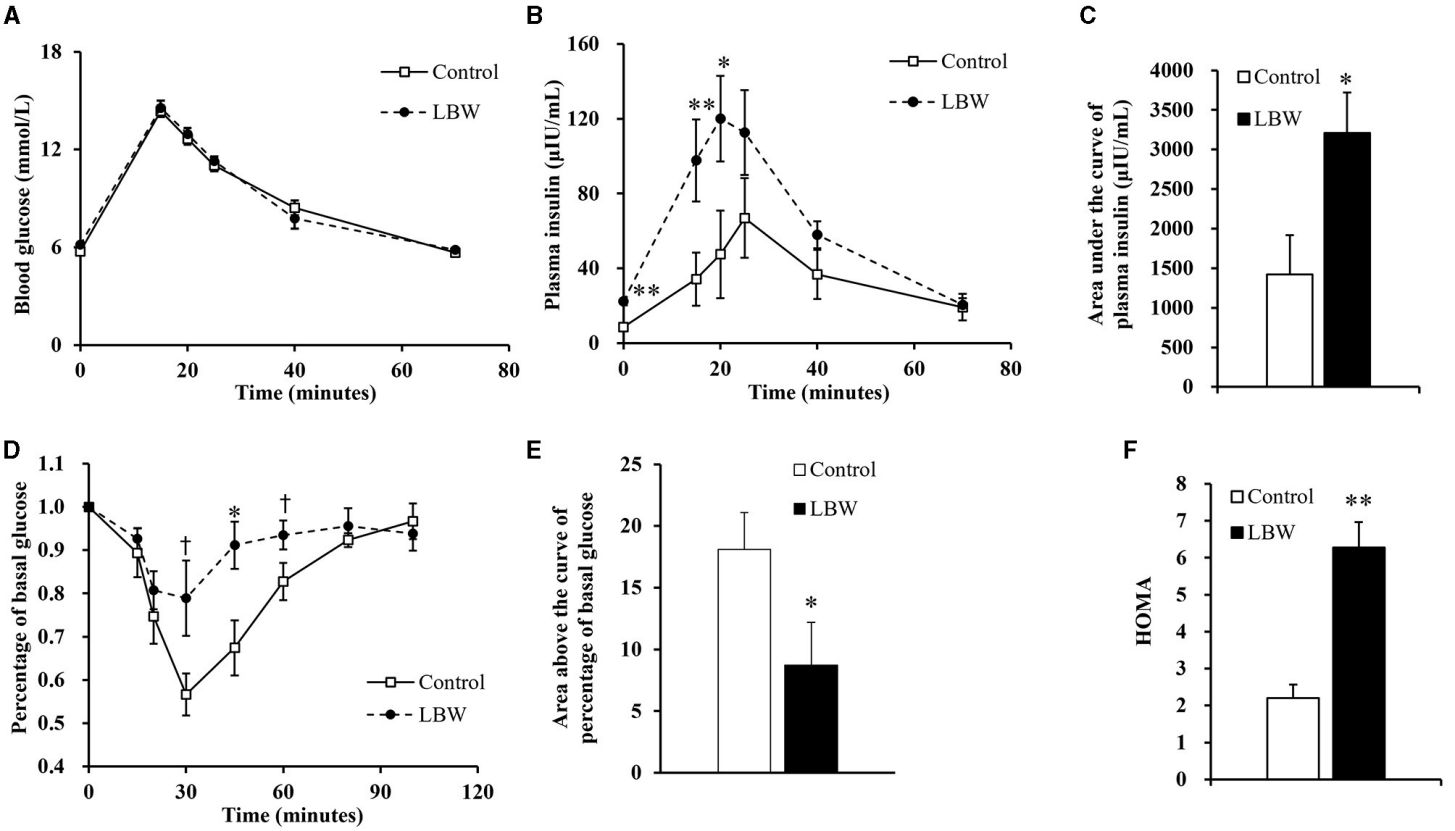
Glucose and insulin tolerance tests. During glucose tolerance test, concentration of blood glucose (*n* = 5) **(A)** was not different from both groups, but plasma insulin (*n* = 5) **(B)** and area under the curve of insulin (*n* = 5) **(C)** were significantly higher in LBW goat kids compared to control. LBW group exhibited lower disposal rate of glucose level (control, *n* = 6; LBW, *n* = 7) **(D and E)** and HOMA **(F)** during insulin tolerance test (*n* = 5).^†^*P* < 0.1; *P < 0.05; **P < 0.01.

During the intravenous glucose tolerance test, the average glucose concentration of LBW goats was similar to that of controls (Figure 2A). In the LBW group, plasma insulin concentration was higher at the first three time points (0, 15, and 20 min, Figure 2B), and the area under the curve of plasma insulin was 2.26-fold higher (*p* < 0.05, Figure 2C). During the insulin tolerance test, glucose concentrations reached the lowest point at 30 min after administering insulin in all animals. The percent of glucose relative to basal was greater (*p* < 0.05, Figure 2D) at 30, 45, and 60 min in LBW goats compared to goats in the control group. Moreover, the area above the curve of glucose percentages was 52% lower (*p* < 0.05, Figure 2E), indicating a decreased disposal rate of glucose levels in LBW goat kids. The marker of insulin resistance, HOMA, was 2.85-fold higher in LBW goats than in NBW goats (*p* < 0.01, Figure 2F).

### 3.3 LBW goat increased free fatty acids in plasma and skeletal muscle

**Figure 3:**
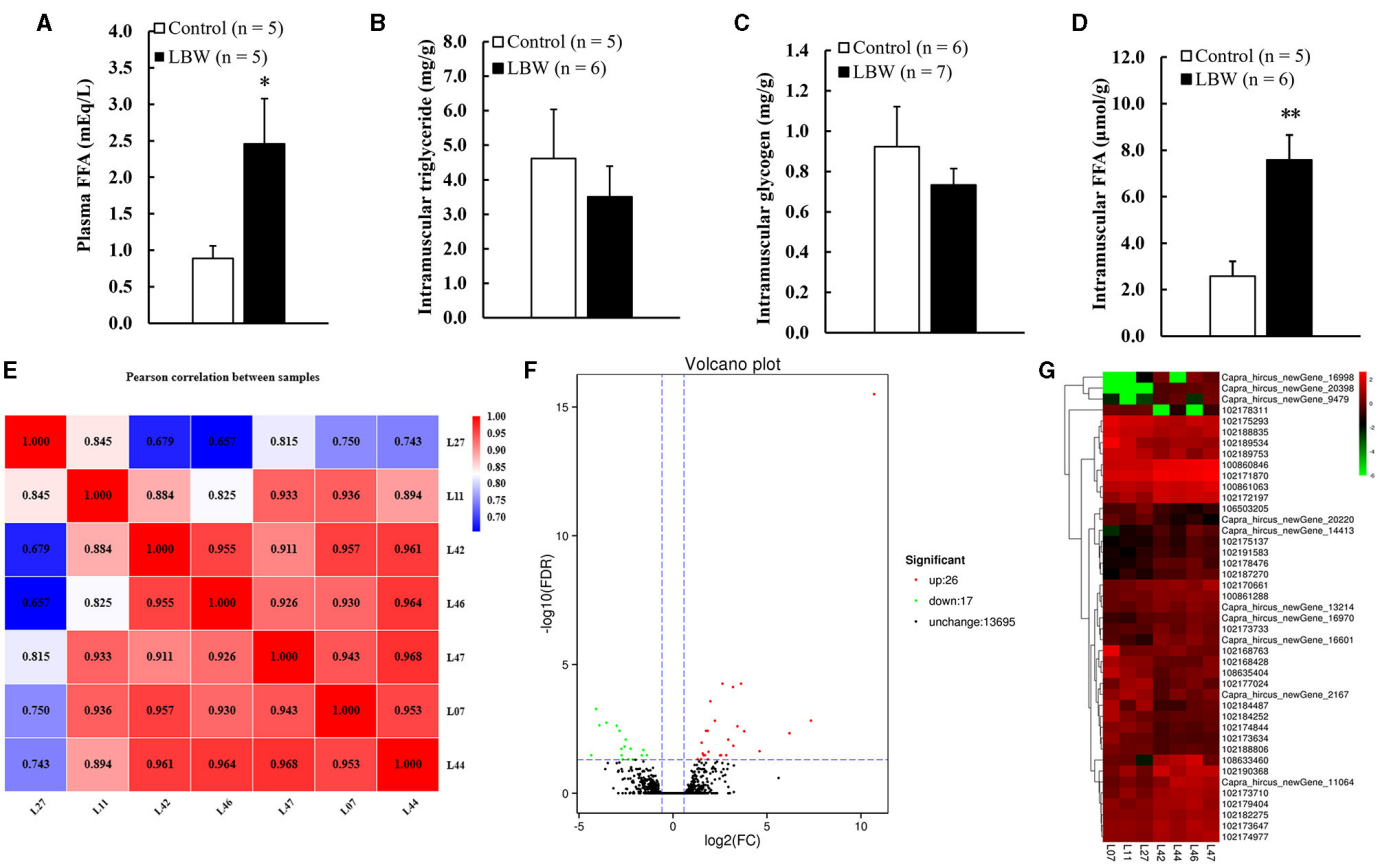
**(A)** Metabolic features of free fatty acids in plasma (*n* = 5); **(B)** intramuscular triglyceride (control, *n* = 5; LBW, *n* = 6); **(C)** intramuscular glycogen (control, *n* = 6; LBW, *n* = 7); **(D)** intramuscular free fatty acids content (control, *n* = 5; LBW, *n* = 6). **(E)** Pearson correlation analysis of all samples (control, *n* = 3; LBW, *n* = 4). **(F)** Volcano plot of global genes expression. **(G)** Cluster heatmap of differential expressed genes in semitendinosus. *P < 0.05; **P < 0.01. FFA, free fatty acids; LBW, low birth weight.

We analyzed the potential variations in FFA, triglycerides, and glycogen levels between groups. The LBW group displayed a substantial increase in plasma FFA (1.8 times, *p* < 0.05) and intramuscular FFA contents (1.9 times, *p* < 0.05) compared to the control. Conversely, there was no significant alteration in the triglyceride and glycogen content (*p* > 0.05, Figures 3B, C). Importantly, the concentrations of glycogen and triglycerides in skeletal muscle remained consistent across the groups.

### 3.4 Transcriptome sequencing data quality assessment of semitendinosus

Seven samples were subjected to a rigorous quality assessment, and the resulting raw sequencing data were meticulously filtered. Detailed data output statistics can be found in Table 2. Following this quality control process, the clean read range for all seven samples fell between 19,423,322 and 9,206,094 base pairs. The GC content exhibited slight fluctuations, ranging from 51.58% to 53.91% in each sample. Notably, the Q30 base percentage exceeded 92%, affirming the reliability of the sequencing data and ensuring its suitability for subsequent experiments.

**Table 2:** Quality assessment of transcriptome sequencing data.

**BMK-ID**	**Clean reads**	**Clean bases**	**GC (%)**	**Q30 (%)**
Control_1	19,423,322	5,812,754,414	52.91	95.47
Control_2	22,855,950	6,844,317,402	52.8	94.98
Control_3	29,206,094	8,744,054,246	50.35	95.14
LBW_1	24,704,868	7,392,310,382	52.19	95.16
LBW_2	21,721,485	6,503,058,008	52.72	95.30
LBW_3	27,166,337	8,123,498,982	52.28	95.17
LBW_4	25,872,950	7,743,884,278	51.58	94.99

GC (%): The percentage of G bases and C bases in the total bases. Q30 (%): The percentage of bases with a mass value ≥Q30.

### 3.5 Differential gene expression analysis in semitendinosus

**Figure 4:**
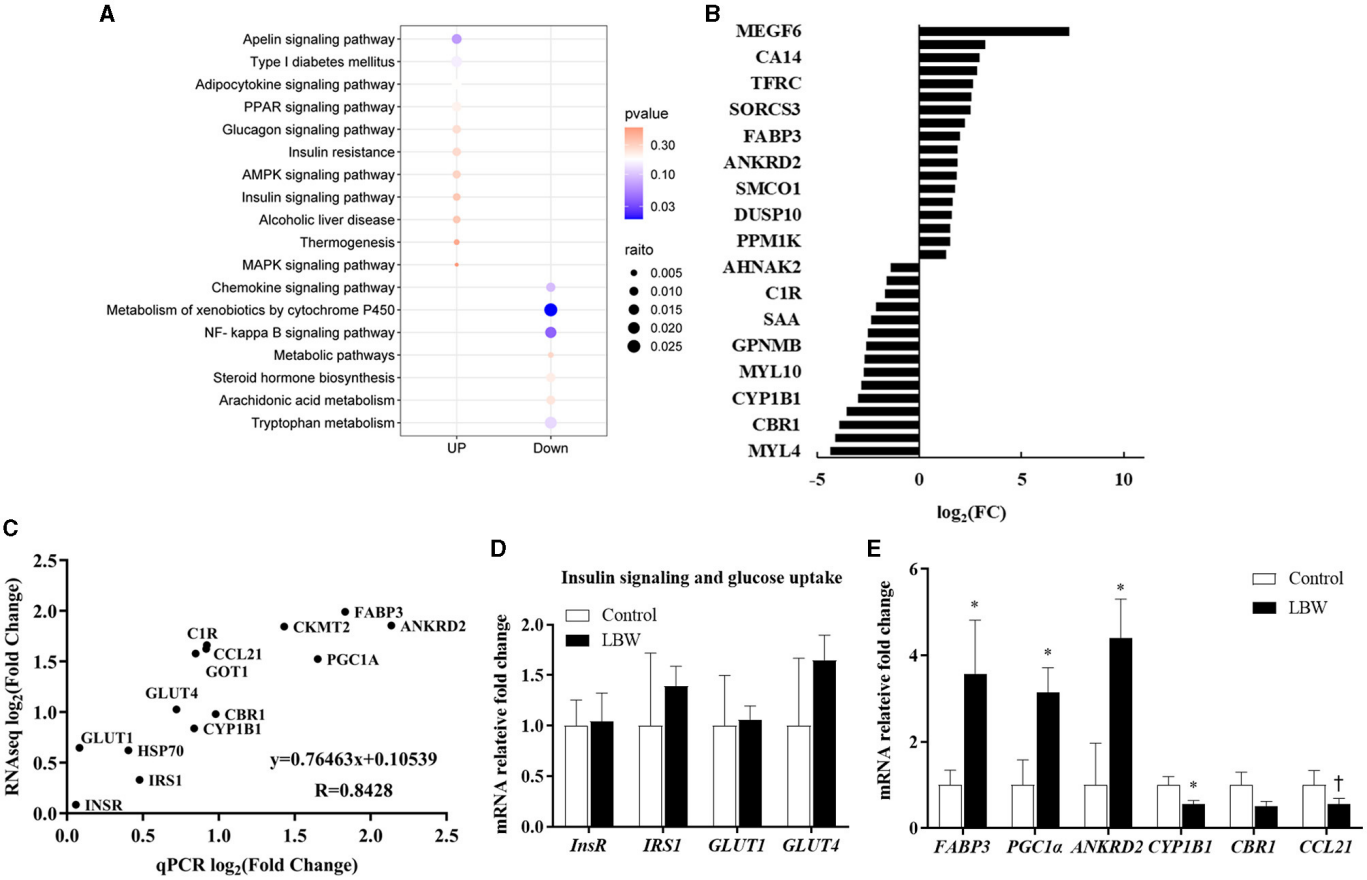
**(A)** KEGG pathway enrichment analysis of DEGs (control, *n* = 3; LBW, *n* = 4). **(B)** Fold changes in the genes with the greatest expression change in skeletal muscle. **(C)** Gene expression determined by RNAseq reflects real-time qPCR (control, *n* = 6; LBW, *n* = 7). **(D)** Differential genes associated with insulin function (control, *n* = 6; LBW, *n* = 7). **(E)** mRNA expression levels in skeletal muscle of control and LBW (control, *n* = 6; LBW, *n* = 7). *P < 0.05;^†^*P* < 0.1.

The DESeq2 software was employed to analyze the transcriptome sequencing data from skeletal muscle samples from both the control and LBW groups. The heatmap showed high correlation indexes in two samples (Figure 3E). The results indicated that, compared to the LBW group, a total of 43 differentially expressed genes were identified, consisting of 26 upregulated and 17 downregulated genes (Figure 3F). Furthermore, a cluster analysis of these 43 differentially expressed genes was undertaken. In addition, differentially expressed genes with unknown functions are presented in Supplementary Table 2. Notably, the results demonstrated that the three replicate samples in the control group and the four replicate samples in the experimental group formed a cohesive cluster, signifying robust biological replication (Figure 3G). In the functional analysis, the differentially expressed genes were predominantly enriched in pathways associated with amino acid metabolism, glucagon and insulin signaling, and fat metabolism, as indicated by the KEGG pathway enrichment analysis (Figure 4A). Specifically, *GOT1* and *CKMT2* were found to be linked to amino acid metabolism pathways, *PGC1*α was associated with glucagon and insulin signaling networks, and *FABP3, CYP1B1, CBR1, CCL21*, and *ANKRD2* were identified as genes relevant to fatty acid metabolism pathways (Table 3).

**Table 3:** Key pathway categories to which differentially expressed genes belong.

**Full gene name**	**Gene symbol**	**Log_2_(FC)**
**Amino acid metabolism**		
Aspartate aminotransferase	GOT1	1.62
Creatine kinase S-type	CKMT2	1.85
**Glucagon and insulin signaling**		
Peroxisome proliferator-activated receptor gamma coactivator 1alpha	PGC1α	1.52
**Fatty acid metabolism and oxidative stress**		
Fatty acid-binding protein	FABP3	1.99
Cytochrome P450 1B1	CYP1B1	−2.99
Carbonyl reductase [NADPH] 1	CBR1	−3.92
C-C motif chemokine 21	CCL21	−1.58
Ankyrin repeat domain-containing protein 2	ANKRD2	1.85

### 3.6 Validation of differentially expressed genes of semitendinosus

In the RNAseq study, the expression of genes that changed the most in the control and LBW groups (upregulated and downregulated) is shown in Figure 4B. To validate the transcriptome sequencing data, a subset of 14 differentially expressed genes (*InsR, IRS1, HSP70, GLUT4, CYP1B1, GOT1, C1R, CCL21, PGC1*α, *FABP3*, and *ANKRD2*) was randomly selected from the 43 differentially regulated genes identified through sequencing (Figure 4C). Each data point represented the mean expression levels derived from RNAseq (control, *n* = 3; LBW, *n* = 4) on the x-axis and the mean expression from real-time qPCR (control, *n* = 6; LBW, *n* = 7) on the y-axis. The correlation coefficient between these data points was 0.84 (*p* < 0.05), indicating a robust correlation between RNAseq and log2 (fold change) values obtained through real-time qPCR. In Figure 4D, it is noteworthy that the expression of regulatory factors associated with insulin function, such as *InsR, IRS1, GLUT1*, and *GLUT4*, remained unaltered under cold stress conditions. However, in Figure 4E, regulatory factors linked to lipid transport and metabolism, specifically *FABP3, PGC1*α, and *ANKRD2*, displayed a significant upregulation in the cold stress group, with fold changes of 3.56, 3.14, and 4.39, respectively (*p* < 0.05).

### 3.7 Low birth weight promotes intramuscular lipid accumulation in goats

**Figure 5:**
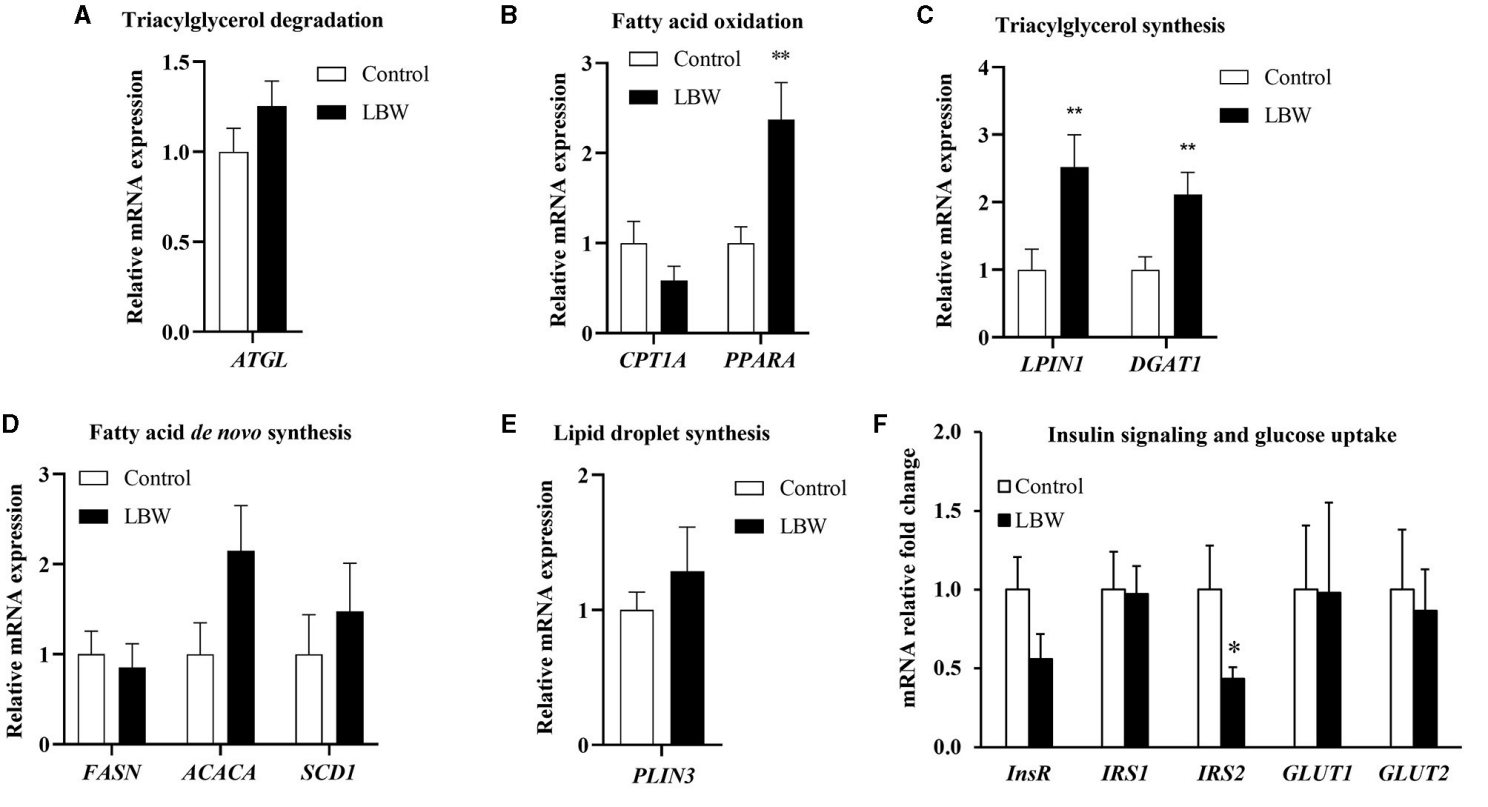
Effects of birth weight on mRNA levels of genes related to fatty acid metabolism. **(A)** Triglyceride decomposition (*ATGL*) (*n* = 4); **(B)** fatty acid oxidation (*CPTI1* and *PPARA*) (*n* = 4); **(C)** triglyceride synthesis (*LPIN1* and *DGAT1*) (*n* = 4); **(D)** *de novo* synthesis of fatty acid *(FASN, ACACA*, and *SCD1*) (*n* = 4); **(E)** lipid droplet synthesis (*PLIN3*) (control, *n* = 4; LBW, *n* = 3). **(F)** Relative fold changes for insulin signaling and glucose uptake pathways in liver (*n* = 5). *P < 0.05; **P < 0.01.

The mRNA expression levels of genes associated with fatty acid *de novo* synthesis (*FASN, ACACA*, and *SCD1*) and of genes involved in triglyceride synthesis (*LPIN1* and *DGAT1*) were assessed using real-time qPCR to investigate the effect of LBW on lipid metabolism in goats. As depicted in Figure 5C, the LBW group exhibited a significant increase in the mRNA expression of *LPIN1* and *DGAT1* compared to the control group (*p* < 0.05). Additionally, we examined the mRNA expression of genes related to triglyceride breakdown (*ATGL*) and fatty acid oxidation (*CPT1A* and *PPARA*). Notably, the experimental group displayed significantly lower levels of *PPARA* mRNA expression when compared to the control group (*p* < 0.05, Figure 5B). There was no significant difference in the mRNA expression of triglyceride breakdown (ATGL), fatty acid *de novo* synthesis and lipid droplet synthesis related genes (*p* > 0.05, Figures 5A, D, E). These findings suggested that LBW in goats may indeed impact critical genes associated with fatty acid metabolism and triglyceride levels.

### 3.8 Impaired insulin signaling in the liver

To further investigate whether lipid accumulation is caused by insulin resistance in the liver, we evaluated factors regulating aspects of insulin signaling and glucose uptake in LBW goat kids. The insulin receptor substrate 2 (*IRS2*) mRNA concentration was 57% lower (*p* < 0.05, Figure 5F). Other mRNA concentrations did not differ in the liver between the two groups (Figure 5F).

## 4 Discussion

Low birth weight is a common problem among newborn goat kids in South China. Previous research has linked LBW to impaired hepatic lipid regulation, but this study is the first to assess insulin-regulated glucose homeostasis in LBW goats during their postnatal development. We found that LBW goat kids had insulin resistance and lower hepatic *IRS2* expression than control goat kids—lipid levels were increased in plasma and the skeletal muscle, and lipid accumulation occurred in the skeletal muscle, indicating impairment of key signaling pathways involved in the regulation of lipid metabolism. These impaired physiological functions and regulations could persist and contribute to metabolic complications in LBW goats later in life.

LBW animals may have an increased risk of metabolic diseases, such as impaired glucose utilization and insulin sensitivity (26), dyslipidemia (5, 15), and oxidative stress (27). Prior to the glucose tolerance test, LBW goat kids had significantly higher fasting plasma insulin concentrations. During the glucose tolerance test, the LBW group also had persistently higher insulin concentrations than the control group (Figure 2B), which almost doubled the area under the curve of insulin (Figure 2C). Furthermore, the insulin tolerance tests showed a lower glucose disposal rate in LBW goat kids (Figures 2D, E) and significantly higher HOMA value. Together, these findings indicate that LBW kids have insulin resistance (Figure 2F).

The liver and skeletal muscle are important organs for glucose uptake in newborn goats, and insulin signaling plays a critical role in glucose uptake and disposal in both the liver and skeletal muscle (28). Previous studies have shown that LBW is closely associated with a compromised insulin-glucose relationship in postnatal life (4, 29, 30). In agreement with these studies, our current study of LBW goat kids showed lower *IRS2* expression in the liver (Figure 5F), but no difference in other regulators of glucose uptake and insulin signaling in the liver or muscle. Mice with complete deletion of hepatic IRS2 develop hyperglycemia, impaired hepatic insulin signaling, and elevated gluconeogenesis (31). Therefore, our finding of lower hepatic *IRS2* could contribute to insulin resistance in LBW newborn goats, as evidenced by the *in vivo* studies.

Commonly, the storage of lipids occurs in adipocytes as intramuscular fat or marbling fleck and within muscle fibers as lipid droplets. The LBW animal model has demonstrated a reduction in insulin-stimulated glucose consumption and augmentation of intramuscular lipids and fatty acids within skeletal muscle (30). In our present study, LBW goat kids exhibited not only an elevated FFA in both plasma and muscle tissue (Figures 3A, D) but also higher mRNA expression levels of the lipid regulatory genes *FABP3* and *PGC1*α in LBW compared to the control group (Figure 4D). FABP3 plays a pivotal role in FFA uptake and transport, with pronounced expression in tissue-specific manners, particularly in the skeletal muscle (31). Moreover, *FABP3* mRNA expression closely correlated with *PGC1*α levels in the skeletal muscle and displayed significant associations with intramuscular fat content in livestock (32). It has been proposed that an upsurge in FFA concentration within the muscle may promote a preference for oxidizing skeletal muscle substrates, potentially competing with glucose, thereby resulting in a sustained inhibition of insulin signal transduction (33). Consequently, disorders and heightened concentrations of intramuscular fatty acids may lead to an elevated risk of dyslipidemia and insulin resistance in LBW goats.

PGC1α plays a pivotal role in regulating energy balance in skeletal muscle (34). Recent studies focusing on LBW have revealed a positive correlation between human birth weight and the muscular expression of *PGC1*α (35). Additionally, when examining muscle-specific PGC1α expression in mice, it was observed that it led to an early increase in the synthesis of FFA, as well as the esterification of fatty acids and the accumulation of triglycerides within skeletal muscle (36). Similar investigations involving the overexpression of muscle-specific *PGC1*α demonstrated an upregulation of gene expression associated with the transfer of fatty acids, notably including FABP3 (37). Consequently, the levels of FFA in LBW animals exhibited a significant increase due to the elevated expression of both *PGC1*α and *FABP3*, contributing to them serving as an energy source for skeletal muscle. In the present study, LBW goat kids exhibited a notable upregulation of *PGC1*α expression within their skeletal muscle. This finding suggests that PGC1α has the potential to regulate insulin sensitivity, which in turn may have a substantial impact on the metabolic stability of skeletal muscle.

Besides compromised energy metabolism, there is compelling evidence suggesting that LBW may detrimentally impact the developmental trajectory of skeletal muscle in animals (38). In a porcine model, LBW pigs exhibited a reduction in both the total number of muscle fibers and muscle cells. Additionally, they demonstrated diminished activity of muscle-specific enzymes, CK and LDH, along with lower protein content when compared to their normal birth weight counterparts in the crib (39). Similarly, in an ovine model, the semitendinosus tissues of LBW lambs manifested decreased DNA content, resulting in a reduction in the number of cell nuclei within the muscle fiber, thereby adversely affecting skeletal muscle development (40). Our current study revealed an upregulation in the expression of *ANKRD2* (Figure 4D), providing evidence for its role in negatively regulating skeletal muscle development, growth, and remodeling (41). ANKRD2, a member of the muscle ankyrin repeat protein family, is implicated in transcriptional responses to mechanical stimulation and stress induced by cellular reactive oxygen species (42). By inhibiting NF-kB transcription, it exerts a negative influence on the expression of various genes associated with muscle inflammatory pathways (41). Consistent with our research, an overexpression of *ANKRD2* in myoblast C2C12 cell lines significantly impacted myogenic cell development, cell fusion, and downregulated muscle-specific regulatory genes such as *MyoD, Myogenin*, and *MYH1* (43). As a result, the mRNA expression level of *ANKRD2* in the skeletal muscle of LBW goats rose, indicating that it negatively affects later muscle growth and development.

The inflammatory response plays a pivotal role in myogenesis by facilitating both muscle fiber damage and regeneration (44). Studies have highlighted the involvement of CC family chemokines in the response to muscle damage and its subsequent repair through their CC chemokine receptors (CCRs) (45). Notably, our RNAseq data revealed lower expression levels of *CCL19* and *CCL21* in the skeletal muscle of LBW individuals (Figure 4D). It is important to note that CCR7's ligands are CCL19 and CCL21. Although the specific role of these chemokines in skeletal muscle was previously unknown, research has implicated CCR2 and CCR5 receptors in chemokine-induced myoblast proliferation and the regulation of muscle injury repair (45). Consequently, the dysregulation of *CCL19* and *CCL21* expression in the skeletal muscle of LBW goats may have an adverse impact on muscular growth following delivery.

## 5 Conclusion

**Figure 6:**
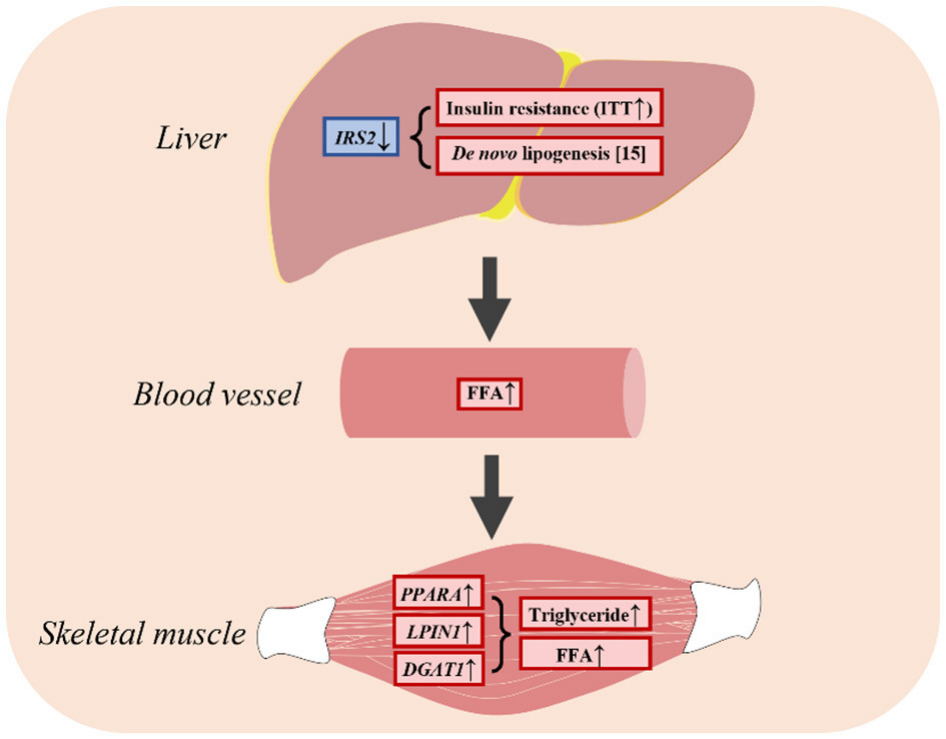
LBW goats contributed to hepatic insulin resistance and had higher FFA in skeletal muscle.

This study showed that insulin resistance promoted lipogenesis by upregulating genes associated with fatty acid uptake, leading to an increase in FFA levels in plasma and the skeletal muscle (Figure 6). As a result, LBW goats might display heightened vulnerability to metabolic disorders during the growth phase. This study provides insights into the characteristics of impaired glucose-insulin metabolism and the underlying factors that could be targeted for early intervention and prevention of metabolic diseases in LBW animals.

## Data availability statement

The original contributions presented in the study are publicly available. This data can be found here: https://www.ncbi.nlm.nih.gov/bioproject/; PRJNA657812.

## Ethics statement

The animal study was approved by the principles and guidelines of the Southwest University Institutional Animal Care and Use Committee (2019, no. GB14925-2010). The study was conducted in accordance with the local legislation and institutional requirements.

## Author contributions

HS: Writing – review & editing. ZH: Writing – review & editing. HF: Writing – review & editing. RL: Writing – review & editing. RZ: Investigation, Writing – review & editing. SL: Conceptualization, Writing – review & editing. YZ: Funding acquisition, Project administration, Writing – review & editing. XC: Conceptualization, Formal analysis, Funding acquisition, Investigation, Project administration, Writing – original draft, Writing – review & editing.

## Supplementary material

Supplementary Figure 1 Verification of RNA quality and integrity by northern blot. In the electrophoresis picture, the left cohort of gel bands was the control, and the right cohort was the low-birth-weight group.

Supplementary Figure 2 Dry matter intake in newborn goats.

Supplementary Table 1 Sequences for primers used in quantitative real-time qPCR.

Supplementary Table 2 List of differentials expressed genes with unknown annotation.
