# Corrigendum: Insulin resistance and dyslipidemia in low-birth-weight goat kids

**DOI:** 10.3389/fvets.2024.1414740

**Published:** 2024-05-08

**Authors:** Huihui Song, Zhuohang Hao, Hehan Feng, Rui Li, Ran Zhang, Sean W. Limesand, Yongju Zhao, Xiaochuan Chen

**Affiliations:** ^1^College of Animal Science and Technology, Southwest University, Chongqing Key Laboratory of Herbivore Science, Chongqing, China; ^2^Yunnan Center for Animal Disease Control and Prevention, Kunming, Yunnan, China; ^3^School of Animal and Comparative Biomedical Sciences, The University of Arizona, Tucson, AZ, United States

**Keywords:** low birth weight, goat, insulin resistance, skeletal muscle, lipid accumulation

In the published article, there was an error in Figure 6 as published. The dyslipidemia caused by insulin resistance is the main reason to induce the higher circulated fatty acids, resulted in accumulation of fatty acids in skeletal muscle. The liver has no direct effect on lipid accumulation in skeletal muscle.

The corrected picture helps the reader understand the article. The corrected Figure 6 and its caption appear below.

**Figure 6:**
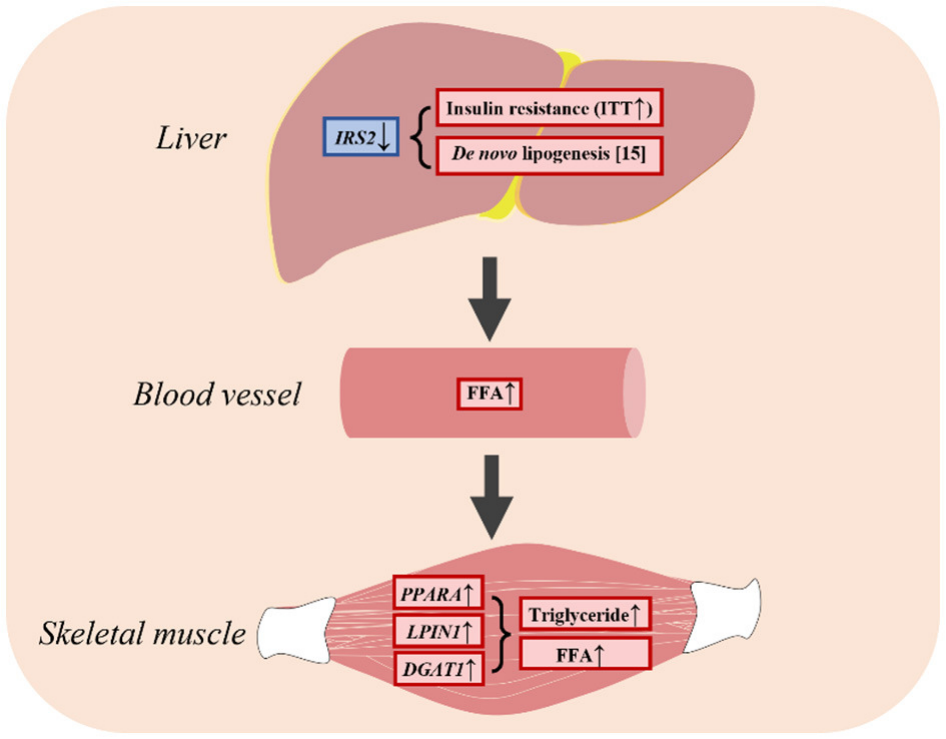
LBW goats contributed to hepatic insulin resistance and had higher FFA in skeletal muscle.

The authors apologize for this error and state that this does not change the scientific conclusions of the article in any way. The original article has been updated.

