# Supplementary figures and images for: Insulin resistance and dyslipidemia in low-birth-weight goat kids
Source: Front Vet Sci. 2024 Mar 26;11:1370640. doi: 10.3389/fvets.2024.1370640 (PMC11002208; doi:10.3389/fvets.2024.1370640)

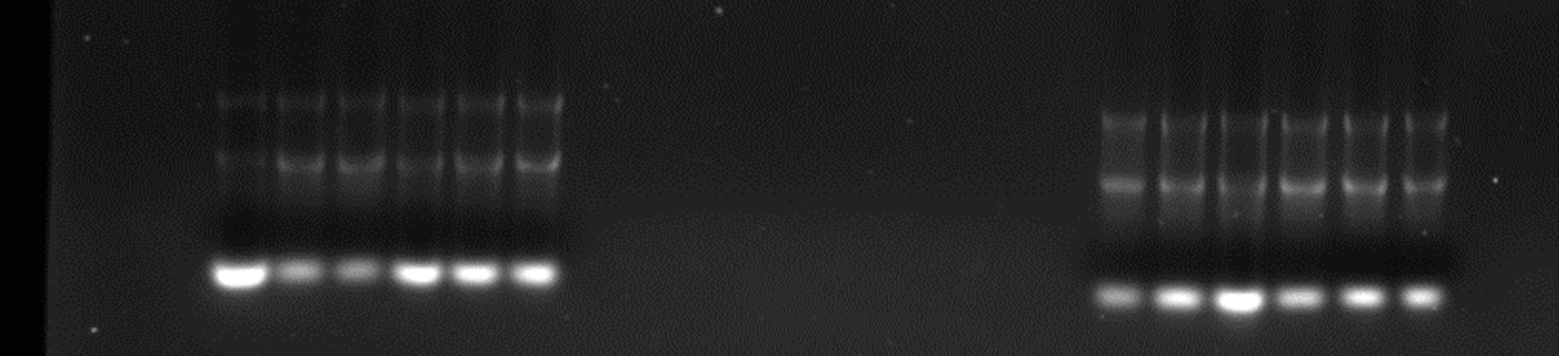

Supplement: Supplementary Figure 1 — Verification of RNA quality and integrity by northern blot. In the electrophoresis picture, the left cohort of gel bands was the control, and the right cohort was the low-birth-weight group. [file Image_1.TIF]

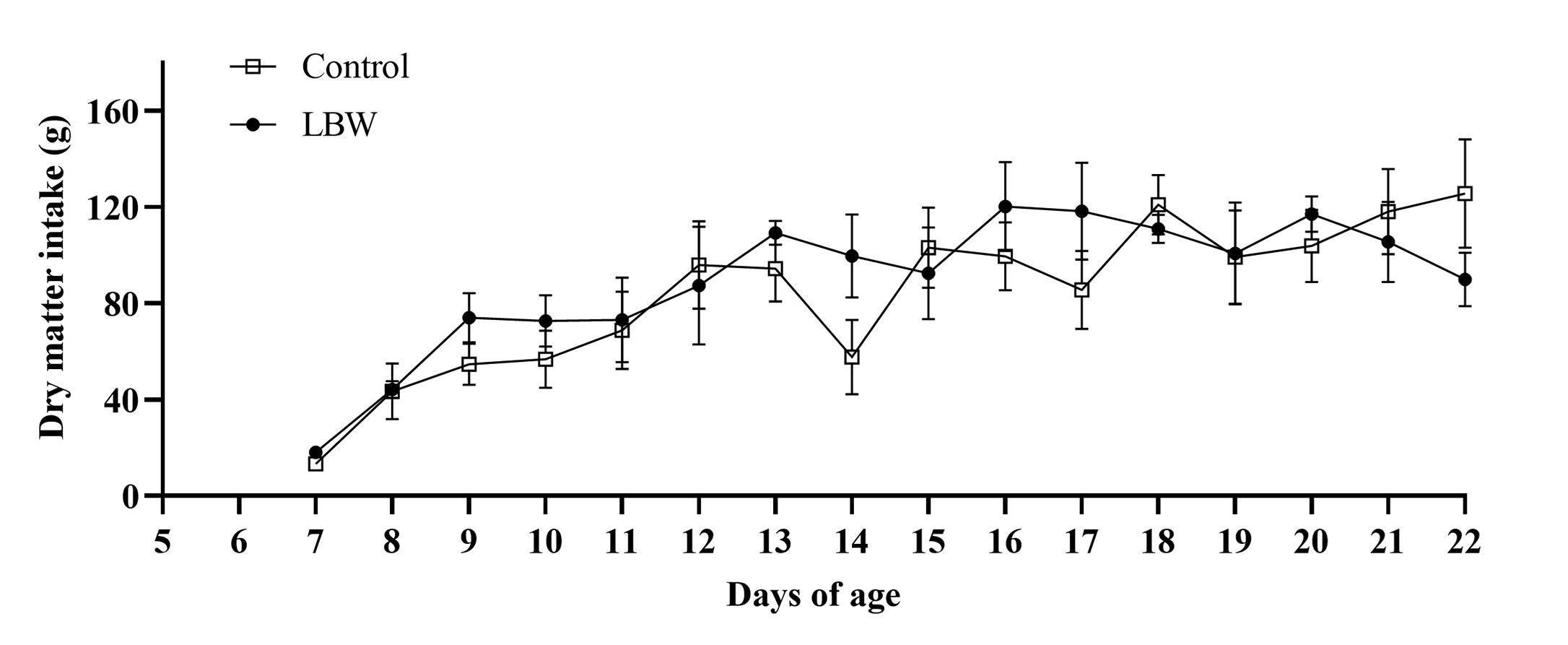

Supplement: Supplementary Figure 2 — Dry matter intake in newborn goats. [file Image_2.TIF]
