# Supplementary material for: Insulin resistance and dyslipidemia in low-birth-weight goat kids
Source: Front Vet Sci. 2024 Mar 26;11:1370640. doi: 10.3389/fvets.2024.1370640 (PMC11002208; doi:10.3389/fvets.2024.1370640)
Supplement: Supplementary Table 1 — Sequences for primers used in quantitative real-time qPCR. [file Table_1.DOCX]

**Table S1** Sequences for primers used in quantitative real-time qPCR.

| **Gene Name** | **Forward Primer (5′→3′)** | **Reverse Primer (5′→3′)** | **Ref** |
| --- | --- | --- | --- |
| GAPDH | TTATGACCACTGTCCACGCC | TCAGATCCACAACGGACACG |  |
| INSR | GTTTTCATCCCCAGGCCTTC | AAGGGTCTGTGCTCCTCTGA |  |
| IRS1 | AGCCCCTCCGACTGCTATTA | GGAGTCGACCGGAACTTGAG |  |
| IRS2 | TTCTCCTCGACCACCACTGT | CGCTGCCTTTCCTGAGAGAG |  |
| GLUT1 | GCCGGACCTTCGATGAGATT | GGAACAGCTCCTCAGGTGTC |  |
| GLUT2 | ACTCAGGGGTGCTATTGGTG | ACATTGGAGAATGGCTGGCA |  |
| GLUT4 | GTTCTTCCTTGGCGCCTACT | CCTAGCACCTGGGCGATTAG |  |
| YWHAZ | AGGAGCCCGTAGGTCATCTT | CAACCTCAGCCAAGTAGCGA |  |
| PGC1A | TGCAACCAGGACTCTGTATGG | TTGGTCACTGCACCACTTGA |  |
| ANKRD2 | CTGCGATCAGTTTCGCCGTA | CCCTCACATTGCTGTCTGCT |  |
| CCL21 | GTCACTGGTCCTGAGCATCC | TCCGAGGCGAGAACAGGATA |  |
| CBR1 | GTCTAGTGATGGGGCTTCAAA | GATGCCAAGTTTCGCCACTC |  |
| CYP1B1 | TTTTTCTGTGGGCAAACGGC | GGCACTCTTTCTCCGCTTGT |  |
| HSP70 | AGTCGGACATGAAGCACTGG | GGTCAGCACCATCAACGAGA |  |
| GOT1 | ATCGCACCGATGATAGCCAG | AGCATTGCACACCTCCTACC |  |
| C1R | TTCTCCAACGAGGACAACGG | GGACGGCAGGAGCAGAAATA |  |
| FABP3 | GTCGGTTTTGCTACCAGGCA | GCCGTGGTCTCATCGAACTC |  |
| CKMT2 | CTATTCCCACCAAGCGCAGA | AGGGTCAAAAAGGTCGGCAA |  |
| ACACA | CTCCAACCTCAACCACTACGG | GGGGAATCACAGAAGCAGCC | [1] |
| SCD1 | CCATCGCCTGTGGAGTCAC | GTCGGATAAATCTAGCGTAGCA | [1] |
| DGAT1 | CCACTGGGACCTGAGGTGTC | GCATCACCACACACCAATTCA | [2] |
| LPIN1 | GAGGGGAAGAAACACCACAA | GTAGCTGACGCTGGACAACA | [3] |
| ATGL | GGAGCTTATCCAGGCCAATG | TGCGGGCAGATGTCACTCT | [4] |
| CPT1A | AAGGACCTCTACGCCAACACG | TTTGCGGTGGACGATGGAG | [5] |
| PPARA | CGGTGTCCACGCATGTGA | TCAGCCGAATCGTTCTCCTAAA | [5] |
| PLIN3 | GGTGGAGGGTCAGGAGAAA | TCACGGAACATGGCGAGT | [5] |
| FASN | GGGCTCCACCACCGTGTTCCA | GCTCTGCTGGGCCTGCAGCTG | [6] |

**Table S2.** List of differentials expressed genes with unknown annotation.

| **Gene ID** | **FDR** | **log_2_FC** |
| --- | --- | --- |
| Capra_hircus_newGene_unknown_1 | 0.018859 | -2.74211 |
| Capra_hircus_newGene_unknown_2 | 0.018765 | -2.25149 |
| Capra_hircus_newGene_unknown_3 | 0.033576 | 1.702647 |
| Capra_hircus_newGene_unknown_4 | 0.00007 | 3.19268 |
| Capra_hircus_newGene_unknown_5 | 0.00252 | 3.430842 |
| Capra_hircus_newGene_unknown_6 | 0.00006 | 3.622495 |
| Capra_hircus_newGene_unknown_7 | 0.003927 | 3.789498 |
| Capra_hircus_newGene_unknown_8 | 0.023294 | 4.607405 |
| Capra_hircus_newGene_unknown_9 | 0.00468 | 6.195321 |
| Capra_hircus_newGene_unknown_10 | 0.00000 | 10.69782 |

Note: The data retained 5 decimal places in this table.

**References**

1. Shi HB, Luo J, Yao DW, Zhu JJ, Xu HF, Shi HP, Loor JJ: **Peroxisome proliferator-activated receptor-γ stimulates the synthesis of monounsaturated fatty acids in dairy goat mammary epithelial cells via the control of stearoyl-coenzyme A desaturase.** *J Dairy Sci* 2013, **96:**7844-7853.

2. Bionaz M, Loor JJ: **Gene networks driving bovine mammary protein synthesis during the lactation cycle.** *Bioinform Biol Insights* 2011, **5:**83-98.

3. Xu H, Luo J, Ma G, Zhang X, Yao D, Li M, Loor JJ: **Acyl-CoA synthetase short-chain family member 2 (ACSS2) is regulated by SREBP-1 and plays a role in fatty acid synthesis in caprine mammary epithelial cells.** *J Cell Physiol* 2018, **233:**1005-1016.

4. Dong XC, Copps KD, Guo S, Li Y, Kollipara R, DePinho RA, White MF: **Inactivation of hepatic Foxo1 by insulin signaling is required for adaptive nutrient homeostasis and endocrine growth regulation.** *Cell Metab* 2008, **8:**65-76.

5. Lin XZ, Luo J, Zhang LP, Wang W, Shi HB, Zhu JJ: **MiR-27a suppresses triglyceride accumulation and affects gene mRNA expression associated with fat metabolism in dairy goat mammary gland epithelial cells.** *Gene* 2013, **521:**15-23.

6. Bionaz M, Loor JJ: **Gene networks driving bovine milk fat synthesis during the lactation cycle.** *BMC Genomics* 2008, **9:**366.
